# Targeting PRMT5/Akt signalling axis prevents human lung cancer cell growth

**DOI:** 10.1111/jcmm.14036

**Published:** 2018-11-20

**Authors:** Shikui Zhang, Yaqiong Ma, Xiaoyan Hu, Yonghua Zheng, Xiaoke Chen

**Affiliations:** ^1^ Department of Emergency People’s Hospital of Gansu Province Lanzhou China; ^2^ Department of Radiology People’s Hospital of Gansu Province Lanzhou China; ^3^ Department of Respiratory Medicine Shanghai Jinshan Tinglin Hospital Shanghai China; ^4^ Department of Thoracic Surgery Shanghai Chest Hospital Shanghai China

**Keywords:** Akt, lung cancer, mTOR, PRMT5, proliferation, PTEN

## Abstract

The emerging evidence reveals that protein arginine methyltransferase 5 (PRMT5) is involved in regulation of tumour cell proliferation and cancer development. Nevertheless, the exact role of PRMT5 in human lung cancer cell proliferation and the underlying molecular mechanism remains largely obscure. Here, we showed that PRMT5 was highly expressed in human lung cancer cells and lung cancer tissues. Furthermore, we generated PRMT5 stable knockdown cell lines (A549 and H1299 cells) and explored the functions of PRMT5 in lung cancer cell proliferation. We found that the down‐regulation of PRMT5 by shRNA or the inhibition of PRMT5 by specific inhibitor GSK591 dramatically suppressed cyclin E1 and cyclin D1 expression and cell proliferation. Moreover, we uncovered that PRMT5 promoted lung cancer cell proliferation via regulation of Akt activation. PRMT5 was directly co‐localized and interacted with Akt, but not PTEN and mTOR. Down‐regulation or inhibition of PRMT5 markedly reduced Akt phosphorylation at Thr308 and Ser473, whereas the expression of PTEN and mTOR phosphorylation was unchanged, indicating that PRMT5 was an important upstream regulator of Akt and induced lung cancer cell proliferation. Altogether, our results indicate that PRMT5 promotes human lung cancer cell proliferation through direct interaction with Akt and regulation of Akt activity. Our findings also suggest that targeting PRMT5 may have therapeutic potential for treatment of human lung cancer.

## 1 INTRODUCTION

Non‐small cell lung cancer (NSCLC), a highly lethal and aggressive malignant tumour, is the most common type of human lung cancer. The NSCLC is the first leading cause of cancer death and the most frequently diagnosed cancer in China. Surgery, chemotherapy, radiotherapy and combination therapy are the main treatment options for lung cancer patients. Nevertheless, the therapeutic effects of those treatments are still poor and the mortality of NSCLC patients remains unacceptably high.^1^ Most lung cancer patients cannot be cured with currently available therapeutic methods. Thus, looking for new targets for NSCLC therapy is urgently needed.

Protein arginine methyltransferase 5 (PRMT5) is the type II arginine methyltransferase that catalyses the symmetrical dimethylation of protein substrates at the arginine residues. Recently, accumulating evidence has shown that PRMT5 is involved in a variety of biological processes via different signalling axis, such as modification of histones and gene expression, chromatin reconstruction, protein modification, cell cycle progression, cell invasion, cell metabolism and development.^2^ In addition to these functions, PRMT5 plays a pivotal role in regulation of cancer cell growth and transformation. It has been reported that down‐regulation of PRMT5 by RNAi prevented cancer cell proliferation and cell cycle transition through PI3K/Akt signalling pathway^3^ and a large number of corroborating studies have shown that PRMT5 expression level was significantly elevated in various cancers, including gastric,^4^ colorectal,^5^ lung,^3^ lymphoma,^6^ leukaemia^7^ and liver^8^ cancers. However, the molecular mechanism of PRMT5 regulation of PI3K/Akt signalling axis is completely unknown.

In this study, our results revealed that PRMT5 was overexpressed in human lung cancer cell lines and human lung cancer tissues. Stable knockdown of PRMT5 or treatment of PRMT5 specific inhibitor in A549 and H1299 cells dramatically suppressed cell proliferation and decreased cyclin E1 and D1 expression. Additionally, PRMT5 promoted lung cancer cell proliferation through direct interaction with Akt and regulation of Akt activity, but not interaction with PTEN and mTOR. Our findings suggest that PRMT5 is a key regulator for human lung cancer cell proliferation and identifying PRMT5 downstream targets will aid in illustrating the role of PRMT5 in human lung cancer development.

## 2 MATERIALS AND METHODS

### 2.1 Cell culture and chemicals

Primary human foetal lung fibroblast cells (IMR90, normal cells) were cultured in RPMI1640 medium with 10% (v/v) foetal bovine serum (FBS, Sigma, cat# F2442). Human lung adenocarcinoma cells A549, H1299, H322, H441 and PC14 were used in this study. A549 cells were cultured in F12K medium/Dulbecco’s Modified Eagle’s Medium (1:1) (cat no. 11320033; Thermo Fisher Scientific) supplemented with 10% (v/v) FBS (Sigma) and the other cells were cultured in RPMI 1640 medium (cat no. 11875119; Thermo Fisher Scientific) with 10% FBS. All cells were maintained at 37°C in a humidified atmosphere containing 5% CO_2_% and 95% air. PRMT5 specific inhibitor GSK591 (cat no. SML‐1751) was purchased from Sigma.

### 2.2 Plasmids and lentivirus preparation

Human PRMT5 and Akt shRNA knockdown lentiviral constructs were generated using pLVTHM vector, and lentivirus were generated by co‐transfection along with packaging plasmid MD2G (Addgene) and helper plasmid PAX2 (Addgene) into HEK293T cells. After 48 hours, the medium was collected and viral titers were pre‐determined. In all indicated experiments, the same number of viral particles was used. Human PRMT5 knockdown targeting sequences are: shRNA‐1,5’‐GGATAAAGCTGTATGCTGT‐3’; shRNA2: 5’‐GCCATCTATAAATGTCTGCTA‐3’. Human Akt knockdown targeting sequences is: 5’‐CCCGAGGTGCTGGAGGACA‐3’.

### 2.3 Knockdown of PRMT5 in lung cancer cells

A549 and H1299 cells were seeded in 6‐well plate for 24 hours before transduction. The cells were infected with lentivirus containing human PRMT5 shRNA or scramble shRNA for 48 hours and then the cells were selected with puromycin (1 µg/mL, cat no. p9620; Sigma) and the non‐infected cells were killed. The stable depletion of PRMT5 cell lines was subjected to next experiments.

### 2.4 Gene expression assay

Total RNA was isolated from A549 and H1299 cells or human lung cancer tissues using TRIzol reagent (Cat no. 15596‐018; Invitrogen) according to the manufacturer’s recommendation, and the equal amount of RNA was subjected to reverse transcription. Quantitative real‐time PCR (qRT‐PCR) was performed with SYBR green fluorescent Dye (cat no. 1725272; Bio‐Rad) with an ABI7800 PCR machine (Applied Biosystems), and GAPGH was used as an internal control. Relative mRNA expression was determined by the ΔΔ−Ct method. Primer sequences are: human PRMT5 forward: 5’‐CCTGTGGAGGTGAACACAGT‐3’ and revise: 5’‐AGAGGATGGGAAACCATGAG‐3’; GAPDH, forward: 5’‐GAAGGTGAAGGTCGGAGTCAACG‐3’ and revise: 5’‐TGCCATGGGTGGAATCATATTGG‐3’.

### 2.5 Cell proliferation assay

For cell proliferation assay, the A549 and H1299 cells were infected with lentivirus containing PRMT5 shRNA or scramble shRNA and seeded into 24‐well plates (5000 cells/plate). Cell proliferation was assessed by cell counting kit‐8 (CCK‐8; Dojindo Molecular Technologies, Rockville, MD, USA) at the indicated time points according to the manufacturer’s protocol. Cell proliferation detection was determined by absorbance at 450 nm using an Infinite 200 plate reader (TECAN, Mönnedorf, Switzerland). To evaluate the effect of PRMT5 inhibitor GSK591 on cell proliferation, A549 and H1299 cells were seeded into 96‐well plates (3000 cells/plate) and treated with vehicle and GSK591 at the indicated concentration and the cell proliferation was measured by CCK‐8.

### 2.6 Western blotting analysis

Proteins were extracted from A549, H1299, H322, H441 PC14 and IMR90 cells and human lung cancer tissues using lysis buffer (20 mmol/L Tris, PH 7.4, 150 mmol/L NaCl, 2 mmol/L EDTA, 2 mmol/L EGTA, 1 mmol/L sodium orthovanadate, 50 mmol/L sodium fluoride, 1% Triton X‐100, 0.1% SDS and 100 mmol/L phenylmethylsulfonyl fluoride) and the extracted proteins were separated in SDS‐polyacrylamide gels and transferred to PVDF membranes. The membranes were washed twice for 5 minutes with PBST and incubated with the PRMT5 (cat no. sc‐376937; Santa Cruz Biotechnology), cyclin E1 (cat no. 20808; Cell Signaling Technology), cyclin D1 (cat no. 2978; Cell Signaling Technology), Akt (cat no. 4691; Cell Signaling Technology), p‐Thr308‐Akt (cat no. 13038; Cell Signaling Technology), p‐Ser473‐Akt (cat no. 4060; Cell Signaling Technology), GSK3β (cat no. 12456; Cell Signaling Technology), p‐Ser9‐GSK3β (cat no. 5558; Cell Signaling Technology), GSK3α (cat no. 4337; Cell Signaling Technology), p‐Ser21‐GSKα (cat no. 9316; Cell Signaling Technology), PTEN (cat no. 9559; Cell Signaling Technology), p‐mTOR‐Ser2448 (cat no. 5536; Cell Signaling Technology), total mTOR (cat no. 2972; Cell Signaling Technology) and Tubulin (cat no. ab6046; Abcam) antibodies at 4°C overnight. After incubation, the membranes were labelled with goat anti‐mouse conjugated to HRP or goat anti‐rabbit conjugated to HRP secondary antibodies (cat no. sc‐2004 and sc‐2005; Santa Cruz Biotechnology). Data were analysed using LI‐COR Image Studio Software (LI‐COR, Biosciences, Lincoln, NE, USA).

### 2.7 Co‐immunoprecipitation

To examine the endogenous interaction between PRMT5 and Akt, A549 and H1299 cells were seeded into 10 cm plates and infected with lentivirus containing PRMT5, Akt or scramble shRNA. Proteins were extracted from these cells using lysis buffer (20 mmol/L Tris, PH 7.4, 150 mmol/L NaCl, 2 mmol/L EDTA, 2 mmol/L EGTA, 1 mmol/L sodium orthovanadate, 50 mmol/L sodium fluoride, 1% Triton X‐100, 0.1% SDS and 100 mmol/L phenylmethylsulfonyl fluoride). The cell lysates were pre‐cleared with protein A or protein G beads at 4°C for 1 hour. Next, 5 µg IgG, PRMT5 and Akt antibody were added into the cell lysates and incubated at 4°C overnight. Subsequently, protein A or protein G beads were added into the cell lysates and incubated at 4°C for 3 hours and then the beads were washed three times with wash buffer (100 mmol/L NaCl, 50 mmol/L Tris pH 7.5, 0.1% NP‐40, 3% glycerol, 100 mmol/L phenylmethylsulfonyl fluoride). Immunoprecipitates were detected by Western blotting.

### 2.8 Immunofluorescence

A549 and H1299 cells were cultured on glass cover slips and incubated overnight to establish adherence. The cells were fixed with 4% paraformaldehyde for 20 minutes at 37°C, followed by permeabilization in ice‐cold methanol for 10 minutes at −20°C. The cells were incubated in blocking buffer (PBS containing 5% normal goat serum and 0.3% Triton X‐100) for 1 hour at room temperature, followed by incubation with anti‐PRMT5 and anti‐p‐ser473‐Akt antibody (diluted 1:100 in blocking buffer) at 4°C overnight. Cells were washed twice for 5 minutes in PBS, and then incubated for 2 hours with Alexa Fluor 488‐conjugated goat anti‐rabbit secondary antibody (cat no. A‐11034; Thermo Fisher) for p‐ser473‐Akt and Alexa Fluor 568‐conjugated goat anti‐mouse secondary antibody (cat no. A‐11004; Thermo Fisher) for PRMT5 (diluted 1:500 in blocking buffer) at room temperature. Nuclei were stained with DAPI (cat no. D9542; Sigma) for 10 minutes at room temperature before observation. The images were captured with Nikon Eclipse E600 fluorescence microscope.

### 2.9 In vitro kinase assay for Akt activity

A549 and H1299 cells were seeded into 10 cm culture dish and infected with lentivirus containing PRMT5 shRNA or scramble shRNA. After 48 hours, the cells were selected with puromycin and the stable depletion of PRMT5 cells were subjected to Akt kinase activity assay by Western blot analysis. The Akt kinase activity was detected by an Akt kinase assay kit (non‐radioactive) according to the manufacturer’s instructions (cat no. 9840; Cell Signaling Technology).

### 2.10 Statistical analysis

All experiments were carried out in triplicate under identical conditions and data were represented as means ± SEM Differences between two groups were analysed by unpaired two‐tailed Student’s *t* test. Difference with *P* < 0.05 was considered statistically significant.

## 3 RESULTS

### 3.1 PRMT5 is highly expressed in human lung cancer cells and tissues

**Figure 1:**
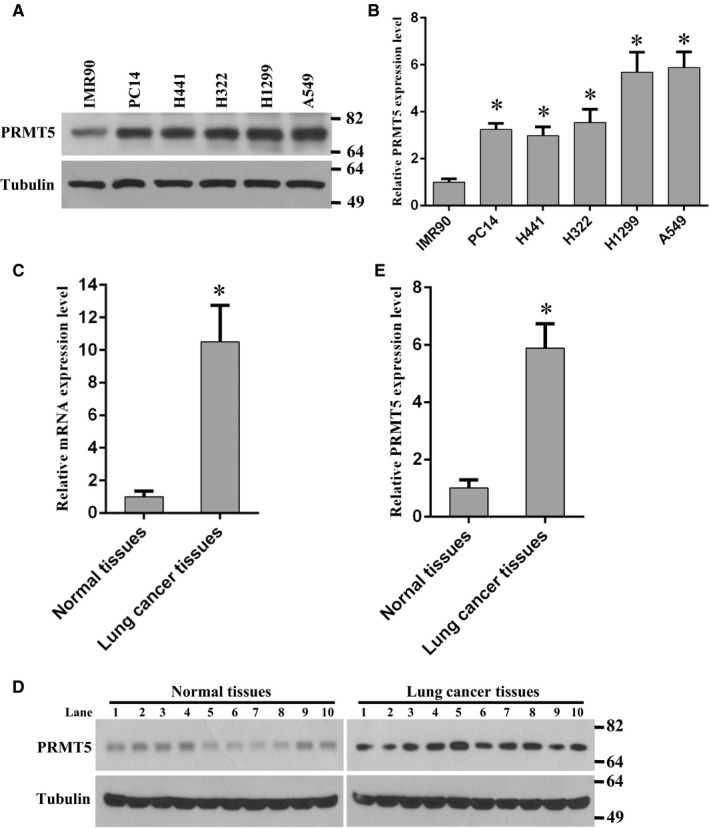
PRMT5 is overexpressed in human lung cancer cells and tissues. (A) PRMT5 protein expression level was detected by Western blotting in different human lung cancer cell lines compared with normal human foetal lung fibroblast cells (IMR90). (B) Quantitative analysis of PRMT5 protein expression level in different human lung cancer cell lines compared with IMR90. *P < 0.05 vs IMR90. (C) PRMT5 mRNA expression level was detected by qRT‐PCR in normal tissues and lung cancer tissues. *P < 0.05 vs normal tissues. (D) PRMT5 protein expression level was determined by Western blotting in normal tissues and lung cancer tissues. (E) Quantitative analysis of PRMT5 protein expression level in normal tissues and lung cancer tissues. *P < 0.05 vs normal tissues

To investigate the functions of PRMT5 in human lung cancer, we firstly examined the PRMT5 protein expression level in different human lung cancer cell lines. As shown in Figure 1A,B, PRMT5 was overexpressed in human lung adenocarcinoma cell lines compared with normal human foetal lung fibroblast cells (IMR90). This result suggests that PRTM5 is involved in human lung tumorigenesis. In order to further confirm our results, the human lung cancer tissues and adjacent normal tissues were used to detect PRMT5 mRNA and protein expression level. As shown in Figure 1C‐E, PRMT5 mRNA and protein expression level was markedly increased in lung cancer tissues compared with normal lung tissues. Taken together, these results imply that PRMT5 plays a pivotal role in human lung cancer progression.

### 3.2 Down‐regulation of PRMT5 prevents lung cancer cell proliferation

**Figure 2:**
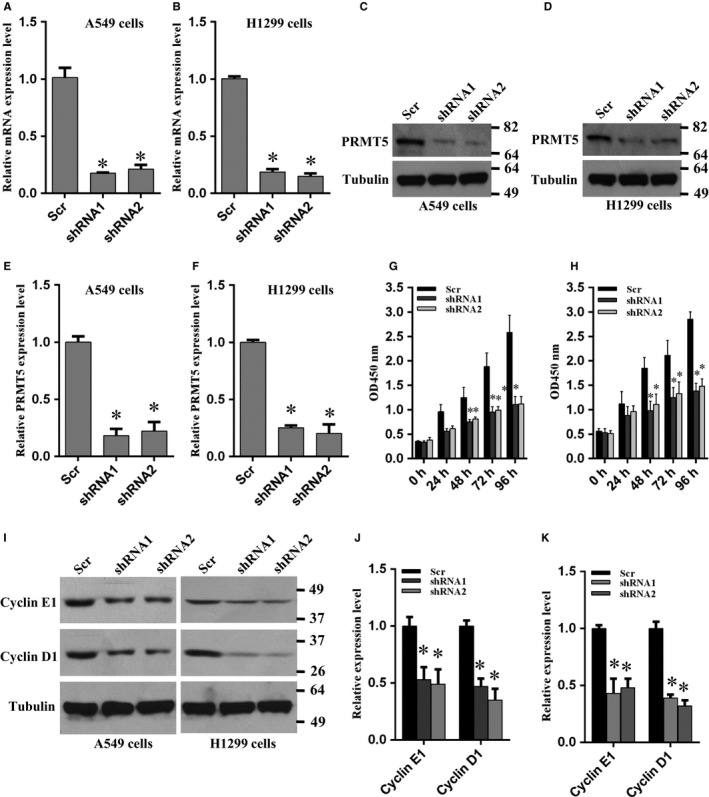
Knockdown of PRMT5 suppresses proliferation of lung cancer cells. (A, B) A549 and H1299 cells were infected with lentivirus containing PRMT5 and scramble (scr) shRNA and PRMT5 mRNA expression level was measured by qRT‐PCR. *P < 0.05 vs scr. (C, D) A549 and H1299 cells were infected with lentivirus containing PRMT5 and scramble (scr) shRNA and the PRMT5 protein expression level was detected by Western blotting. (E, F) Quantitative analysis of PRMT5 protein expression level in A549 and H1299 cells. *P < 0.05 vs scr. (G, H) A549 and H1299 cells were infected with lentivirus containing PRMT5 and scramble (scr) shRNA and the cell proliferation were measured by CCK‐8 assay at the indicated time points. *P < 0.05 vs scr. (I) The cyclin E1 and cyclin D1 expression level was detected by Western blotting when PRMT5 was down‐regulated in A549 and H1299 cells. (J, K) Quantitative analysis of cyclin E1 and cyclin D1 protein expression level in A549 and H1299 cells. *P < 0.05 vs scr

To investigate whether PRMT5 is implicated in lung cancer cell proliferation, we delivered the PRMT5 and scramble shRNA into A549 and H1299 cells by lentivirus and generated PRMT5 stable knockdown cells. As shown in Figure 2A,B, the PRMT5 mRNA expression level was significantly reduced both in A549 and H1299 cells compared with scramble group. We also detected PRMT5 protein expression level by Western blotting. As shown in Figure 2C‐F, PRMT5 protein expression level was markedly decreased both in A549 and H1299 cells compared with scramble group. Thus, these PRMT5 stable knockdown cells were used for next experiments.

Subsequently, cell proliferation rate was measured in these PRMT5 stable knockdown cells. As shown in Figure 2G,H, cell proliferation was dramatically blocked when PRMT5 was down‐regulated both in A549 and H1299 cells during the different time points. These results indicate that PRMT5 is involved in human lung cancer cell proliferation. Previous study has reported that PRMT5 promoted liver cancer cell growth through inhibiting BTG2 expression and the up‐regulation of cyclin E1 and cyclin D1.^9^ Therefore, we asked if down‐regulation of PRTM5 could reduce cyclin E1 and cyclin D1 expression in lung cancer cells. As expected, cyclin E1 and cyclin D1 expression was significantly decreased both in A549 and H1299 cells when PRMT5 was knocked down (Figure 2I‐K). These results indicate that PRMT5 regulates lung cancer cell proliferation via control of cell cycle progression.

### 3.3 Inhibition of PRMT5 activity suppresses lung cancer cell proliferation

**Figure 3:**
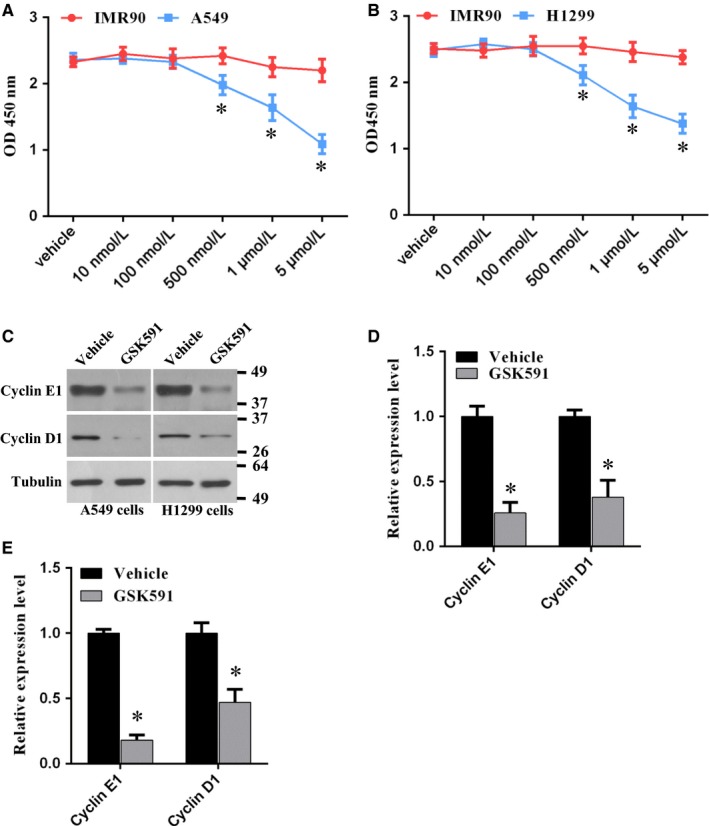
Blocking PRMT5 by GSK591 represses proliferation of lung cancer cell. (A) IMR90 and A549 cells were treated with PRMT5 inhibitor GSK591 or vehicle at the indicated concentrations for 4 days and cell proliferation was measured by CCK‐8 assay. *P < 0.05 IMR90 vs A549 cells. (B) IMR90 and H1299 cells were treated with PRMT5 inhibitor GSK591 or vehicle at the indicated concentrations for 4 days and cell proliferation was measured by CCK‐8 assay. *P < 0.05 IMR90 vs H1299 cells. (C) A549 and H1299 cells were treated with PRMT5 inhibitor GSK591 (1 μmol/L) or vehicle and the cyclin E1 and cyclin D1 protein expression level was detected by Western blotting. (D, E) Quantitative analysis of cyclin E1 and cyclin D1 protein expression level in A549 and H1299 cells. *P < 0.05 vs vehicle

To further investigate the role of PRMT5 in lung cancer cell proliferation, we used PRTM5 specific inhibitor, GSK591, to black PRMT5 activity and cell proliferation was evaluated. We found that proliferation of A549 and H1299 cells was markedly reduced in a dose dependent manner, whereas proliferation of IMR90 cells was almost no change (Figure 3A,B), indicating that this inhibitor has selectivity between cancer cells and normal cells. Next, the cell cycle proteins, cyclin E1 and cyclin D1, in A549 and H1299 cells were assessed upon the treatment of GSK591. We found that cyclin E1 and cyclin D1 expression level was significantly decreased both in A549 and H1299 cells (Figure 3C‐E). Taken together, these results suggest that blocking PRMT5 activity can prevent lung cancer cell proliferation and cell cycle progression.

### 3.4 PRMT5 interacts with Akt in lung cancer cells

**Figure 4:**
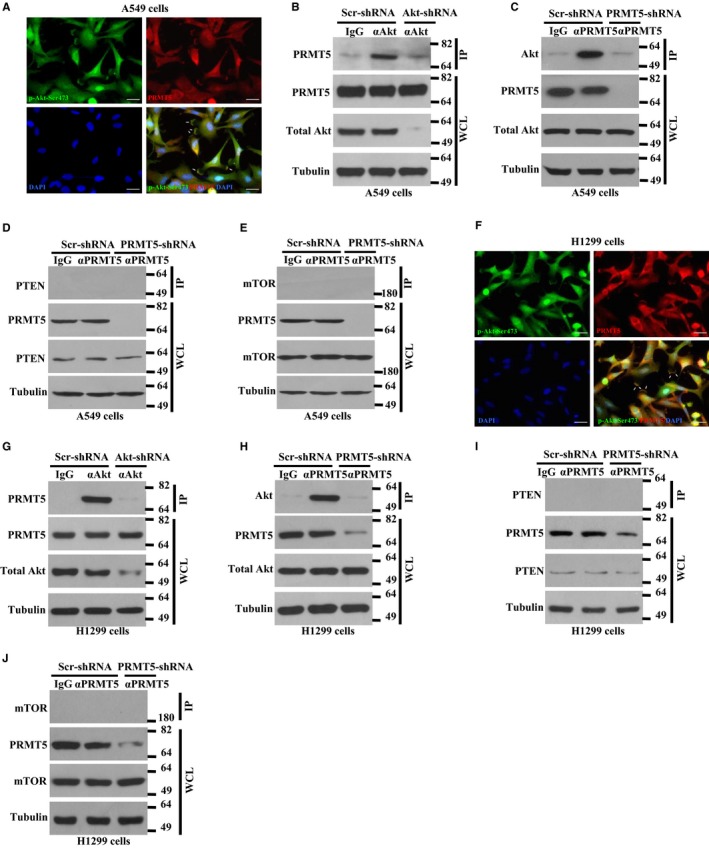
PRMT5 directly interacts with Akt in lung cancer cells. (A) The co‐localization of endogenous PRMT5 and p‐Akt‐Ser473 (activated Akt) were shown in A549 cells using immunofluorescence. (B) A549 cells were infected with lentivirus containing Akt and scramble shRNA and cell extracts from A549 cells were immunoprecipitated with an antibody against Akt and immunoblotted with an antibody against PRMT5. (C) A549 cells were infected with lentivirus containing PRMT5 and scramble shRNA and cell extracts from A549 cells were immunoprecipitated with an antibody against PRMT5 and immunoblotted with an antibody against Akt. (D) A549 cells were infected with lentivirus containing PRMT5 and scramble shRNA and cell extracts from A549 cells were immunoprecipitated with an antibody against PRMT5 and immunoblotted with an antibody against PTEN. (E) A549 cells were infected with lentivirus containing PRMT5 and scramble shRNA and cell extracts from A549 cells were immunoprecipitated with an antibody against PRMT5 and immunoblotted with an antibody against mTOR. (F) The co‐localization of endogenous PRMT5 and p‐Akt‐Ser473 (activated Akt) were shown in H1299 cells using immunofluorescence. (G) H1299 cells were infected with lentivirus containing Akt and scramble shRNA and cell extracts from H1299 cells were immunoprecipitated with an antibody against Akt and immunoblotted with an antibody against PRMT5. (H) H1299 cells were infected with lentivirus containing PRMT5 and scramble shRNA and cell extracts from H1299 cells were immunoprecipitated with an antibody against PRMT5 and immunoblotted with an antibody against Akt. (I) H1299 cells were infected with lentivirus containing PRMT5 and scramble shRNA and cell extracts from H1299 cells were immunoprecipitated with an antibody against PRMT5 and immunoblotted with an antibody against PTEN. (J) H1299 cells were infected with lentivirus containing PRMT5 and scramble shRNA and cell extracts from H1299 cells were immunoprecipitated with an antibody against PRMT5 and immunoblotted with an antibody against mTOR

PRMT5 plays an important role in cancer cell growth and proliferation, especially in human lung cancer cells. However, the underlying molecular mechanism is completely unknown. Previous study has reported that PRMT5 up‐regulated cyclin‐dependent kinase and PI3K/Akt signalling pathway.^3^ Nevertheless, this study only performed ectopic expression of PRMT5 in 293T cells and there was no information about how PRTM5 regulated PI3K/Akt signalling pathway. To investigate whether PRMT5 directly interacted with Akt, immunofluorescence was used to observe the co‐localization between PRMT5 and activated Akt. As shown in Figure 4A, endogenous PRMT5 (Red) was co‐localized with activated Akt (p‐Ser473‐Akt, green) in the cell surface in A549 cells. This result indicates that PRMT5 may directly regulate Akt activity. If PRMT5 indeed regulates Akt activation, PRMT5 may directly interact with Akt. To this end, the co‐immunoprecipitation was used to determine whether PRMT5 directly interact with endogenous Akt in A549 cells. As shown in Figure 4B, PRMT5 was strongly interacted with Akt, but this interaction was significantly diminished when Akt was down‐regulated by shRNA. Similarly, Akt was strongly interacted with PRMT5 as well, but the interaction was dramatically impaired when PRMT5 was down‐regulated by shRNA (Figure 4C). We also performed the co‐immunoprecipitation between PRMT5 and PTEN or mTOR, respectively. However, there is no interaction between PRMT5 and PTEN or mTOR (Figure 4D,E). Similar results were obtained from H1299 cells, including co‐localization (Figure 4F) and interaction (Figure 4G‐J) between PRMT5 and Akt. Collectively, these results demonstrate that PRMT5 directly interact with Akt but no PTEN or mTOR in human lung cancer cells.

### 3.5 PRMT5 regulates Akt activation in lung cancer cells

**Figure 5:**
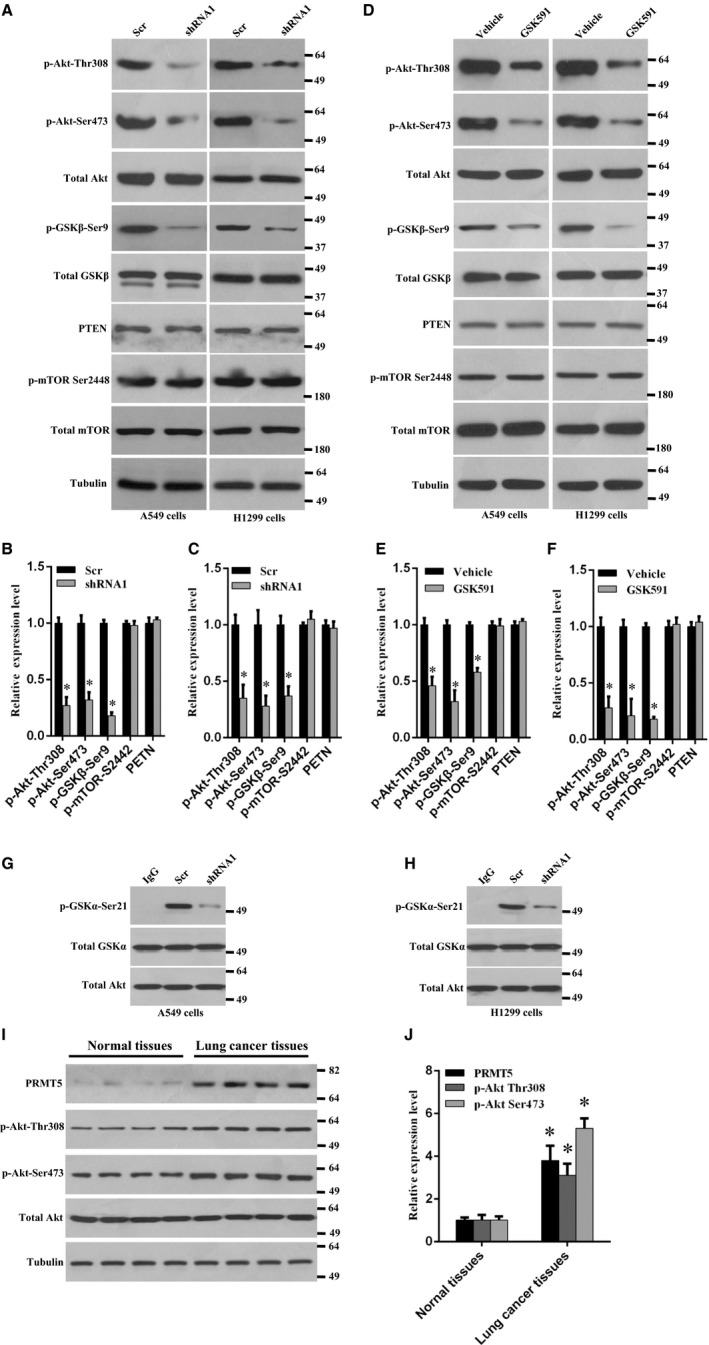
PRMT5 regulates Akt activation in lung cancer cell. (A) A549 and H1299 cells were infected with lentivirus containing PRMT5 and scramble shRNA and the indicated proteins were detected by Western blotting. (B, C) Quantitative analysis of indicated proteins expression level in A549 and H1299 cells. *P < 0.05 vs scr. (D) A549 and H1299 were treated with PRMT5 inhibitor GSK591 (1 μmol/L) or vehicle for 4 days and indicated proteins were detected by Western blotting. (E, F) Quantitative analysis of indicated proteins expression level in A549 and H1299 cells upon PRMT5 inhibitor stimulation. *P < 0.05 vs vehicle. (G, H) A549 and H1299 cells were infected with lentivirus containing PRMT5 and scramble shRNA and the kinase activity of Akt was evaluated by in vitro kinase assay kit. (I) Western blotting analysis of indicated proteins expression level from paired human lung cancer tissues and adjacent normal tissues. (J) Quantitative analysis of indicated proteins expression level in human lung cancer tissues and normal tissues. *P < 0.05 vs normal tissues

PI3K/Akt signalling pathway is essential for cell proliferation, especially in many human cancer types.^10^ We wonder whether PRMT5 regulates Akt activation in human lung cancer cells. As shown in Figure 5A‐C, Akt phosphorylation (Thr308 and Ser473) and the Akt downstream target GSK3β phosphorylation (Ser9) was markedly decreased when PRMT5 was knocked down both in A549 and H1299 cells, but PTEN and mTOR phosphorylation (Ser2442) did not change. We also examined the effect of PRMT5 inhibitor GSK591 on the Akt activity. As shown in Figure 5D‐F, Akt phosphorylation (Thr308 and Ser473) and GSK3β phosphorylation (Ser9) was significantly reduced when A549 and H1299 cells were treated with GSK591, whereas the PTEN and mTOR phosphorylation (Ser2442) did not change, indicating that PRMT5 regulation of Akt activity seems to be independent of PTEN and mTOR. To further confirm our results, the in vitro Akt kinase assay was performed both in A549 and H1299 cells. As shown in Figure 5G,H, the GSK3α phosphorylation (Ser21) was strikingly reduced when PRMT5 was down‐regulated by shRNA compared with scramble group, indicating that Akt activity was impaired and PRMT5 is required for Akt activation. Collectively, these results demonstrate that PRMT5 promotes lung cancer cell proliferation through regulation of Akt activity, but not PTEN and mTOR signalling pathway.

Finally, we evaluated the PRMT5 expression level and Akt activity in human lung cancer tissues and adjacent normal tissues. As shown in Figure 5I,J, PRMT5 expression level was markedly up‐regulated in lung cancer tissues compared with normal tissues. Moreover, concomitant with up‐regulation of PRMT5 protein expression level, the Akt phosphorylation (Thr308 and Ser473) was also dramatically enhanced. This result further implies that PRMT5 is participated in human lung cancer progression through the regulation of Akt activity.

## 4 DISCUSSION

To date, it is clear that PRMT5 is an oncoprotein and plays a pivotal role in various human cancer progressions through the regulation of different signalling cascades. However, it is still unclear whether PRMT5 interacts with Akt, another important oncoprotein in human cancers, and regulates Akt activity in human lung cancer. The findings in this study showed that PRMT5 was overexpressed in human lung cancer cells (Figure 1). This phenomenon was further assessed in the analysis of human lung cancer tissues, in which we detected ectopic expression level of PRMT5 in human lung cancer tissues and very low expression level of PRMT5 in adjacent lung tissues (Figure 1). In addition, we uncovered that down‐regulation of PRMT5 by shRNA or blocking PRMT5 activity by specific inhibitor markedly prevented proliferation of A549 and H1299 cells (Figures 2 and 3). Our findings also revealed that PRMT5 was directly co‐localized and interacted with Akt and PRMT5 regulated Akt kinase activity both in A549 and H1299 cells (Figures 4 and 5). These results implied that PRMT5 expression level and its downstream targets could be served as novel candidates for human lung cancer therapy. Our findings raise the possibility that PRMT5 promotes human lung cancer cell proliferation via direct interaction and regulation of Akt activation.

Previous study has reported that silencing PRMT5 arrested cell proliferation at G1 phase.^11^ In addition, CDK4/cyclin D1 complex regulated PRMT5 kinase activity by phosphorylation of MEP50^12^ and PRMT5 promoted liver cancer cell growth through inhibiting BTG2 expression and up‐regulation of cyclin E1 and cyclin D1.^9^ Furthermore, CDT1 was methylated and stabilized by PRMT5, which promoted cells entry into S phase.^12^ Transcriptional factor E2F, a key cell cycle regulator, was involved in cell‐cycle progression and led to cells entry from G1‐ to S‐phase.^13^ E2F1 was directly methylated by PRMT5 and the methylation of E2F1 by PRMT5 was required for regulating E2F1’s biochemical and functional properties,^14^ indicating that arginine methylation resulted in cell‐cycle progression affected by E2F1. All these observations suggest that PRMT5 is essential for cell proliferation via regulation of cell cycle progression and that PRMT5 is an important upstream effector for cancer cell growth. In this study, we showed that cyclin E1 and cyclin D1 expression was significantly decreased in both A549 and H1299 cells when PRMT5 was down‐regulated or blocked by GSK591 (Figures 2I and 3C). Our findings not only validate that PRMT5 regulates cells proliferation via control of cell cycle, but also indicate that PRMT5 is a key regulator for human lung cancer cell growth.

Protein kinase B (also named Akt) is required for different cellular processes, from the regulation of cell cycle, survival and growth to control of cell metabolism.^10^ Dysfunction of Akt is implicated in various human cancers, type II diabetes, cardiovascular disease, neurodegenerative disease and autoimmune disorders.^10^ Previously published study has shown that PRMT5 activated Akt by inducing hyperphosphorylation of the upstream positive regulator PI3K and hypophosphorylation of the negative regulator PTEN.^3^ However, this study did not investigate how PRMT5 induces Akt activation and the underlying molecular mechanism is still unknown. On the other hand, there is no evidence that PRMT5 activates Akt in human lung cancer cells, although Wei et  al reported that overexpression of PRMT5 induced Akt activation (phosphorylation) in 293T cells. In this study, we showed that PRMT5 was directly co‐localized and interacted with Akt and that PRMT5 regulated Akt kinase activity both in A549 and H1299 cells (Figures 4 and 5). Wei et  al also reported that GFP‐PRMT5 overexpression dramatically elevated mTOR phosphorylation (S2448), which contributed to regulate Akt activation by PRMT5.^3^ However, we did not observe any difference in PTEN, p‐mTOR and total mTOR expression levels when PRMT5 was down‐regulated by shRNA or PRMT5 activity was blocked by specific inhibitor both in A549 and H1299 cells. Akt activation resulted in phosphorylation of many downstream targets, including mTOR/eIF4E^15^ and GSK3β (Ser9).^16^ EIF4E and GSK3β is the positive and negative effector of cyclin D1, respectively.^17^, ^18^, ^19^, ^20^ That is why PRMT5 can induce cyclin D1 expression through the regulation of Akt/eIF4E and Akt/GSK3β signalling pathways. All these observations indicate that PRMT5 promotes human lung cancer cells proliferation by interaction with Akt and regulation of Akt kinase activity, which further lead to the activation or inhibition of key downstream targets of Akt by phosphorylation.

In summary, our results revealed that PRMT5 was frequently overexpressed in human lung cancer cells and tissues. Silencing PRMT5 or blocking PRMT5 activity prevented lung cancer cell growth and proliferation through induction of cell cycle arrest. Moreover, PRMT5 directly co‐localized and interacted with Akt but not PTEN or mTOR and down‐regulation of PRMT5 decreased Akt phosphorylation and kinase activity. These results strongly imply that PRMT5 may serve as a new therapeutic target for the treatment of human lung cancer. More importantly, the new insights into the activation of Akt medicated by PRMT5 provide much new information for the mechanisms of carcinogenic effect of PRMT5 in human lung cancer.

## CONFLICT OF INTEREST

All the authors declare that they have no conflict of interest.
